# Clinical and Therapeutic Characteristics of Cancer Patients in the Southern Region of Saudi Arabia: A Cross-Sectional Study

**DOI:** 10.3390/ijerph18126654

**Published:** 2021-06-21

**Authors:** Hamad S. Alyami, Abdallah Y. Naser, Eman Zmaily Dahmash, Mohammad H. Alyami, Osamah M. Belali, Ahmad M. Assiri, Amjad Rehman, Abdulrhman M. Alsaleh, Hind A. Alsaleh, Shahad H. Hussein, Shahad M. Amer, Sara A. Asiri, Amjad I. Almuadi

**Affiliations:** 1 Department of Pharmaceutics, College of Pharmacy, Najran University, Najran 66262, Saudi Arabia; mhalmansour@nu.edu.sa; 2 Department of Applied Pharmaceutical Sciences and Clinical Pharmacy, Faculty of Pharmacy, Isra University, Amman 11622, Jordan; abdallah.naser@iu.edu.jo (A.Y.N.); eman.zmaily@iu.edu.jo (E.Z.D.); 3 Pharmaceutical Services Department, Asir Central Hospital, Abha 62529, Saudi Arabia; osamabelali@yahoo.com (O.M.B.); Ahmuassiri@moh.gov.sa (A.M.A.); 4 Oncology Department, Asir Central Hospital, Abha 62529, Saudi Arabia; rehmanamjad@hotmail.com; 5 College of Pharmacy, King Khalid University, Abha 62529, Saudi Arabia; Abdu.1996@icloud.com (A.M.A.); hind_ahmad@hotmail.com (H.A.A.); Shahadhussein041216@gmail.com (S.H.H.); shahad.mya@hotmail.com (S.M.A.); D.ph.sara@gmail.com (S.A.A.); Amjad.assiri28@gmail.com (A.I.A.)

**Keywords:** Asir, cancer, epidemiology, Saudi Arabia

## Abstract

Aims: Due to the continuous changes in modern lifestyle and the need to explore the effect of these changes on the risk of developing cancer, ongoing research on the epidemiology and characteristics of cancer patients is requested. This study explored the epidemiology of cancer, its characteristics, treatment patterns, and risk factors in the southern region of Saudi Arabia. Methods: A retrospective cross-sectional study was conducted using cancer patients’ medical records at Asir Central Hospital in the southern region of Saudi Arabia. Active patients’ records were extracted between January 2013 and December 2019. Results: A total of 2038 patients were identified during the study period, with a mean age of 60.9 (SD: 19.0) years. The patients had survived with their cancer for a median duration of 4 years (IQR: 2–6). Around 4.6% of the patients required ICU admission with a median period of 9 days (IQR: 5–14.75). The death rate during the study period was 10.9%. Around 20.8% of the cases were metastatic, of which 77.8% were at stage four of metastasis, and 19.7% of the patients were receiving chemotherapy for their disease. The most common types of cancer were malignant neoplasms of digestive organs, comprising 40.8% of the sample. Older age (60 years and above) and using specific chronic disease medications were predictors associated with a higher risk of death due to cancer (*p* < 0.05). Smoking history, using specific chronic disease medications, and having previous surgery were predictors associated with a higher risk of ICU admission (*p* < 0.05). Conclusion: Breast, colon, and liver cancers were the most prevalent in the southern region of Saudi Arabia. Several modifiable cancer risk factors were identified. The results of this study should support decision-makers in the initiation of programs for key modifiable risk factors that enhance lifestyle changes and reduce cancer risks.

## 1. Introduction

Due to the continuous changes in popular lifestyle and the need to explore the effect of these changes on the risk of developing cancer, ongoing research on the epidemiology and characteristics of cancer patients have been requested. Having current information on the most common types of cancers enables decision-makers to make informed decisions in terms of resource allocation, planning, and better use of healthcare resources [1]. Additionally, it enables healthcare providers to identify individuals who have a higher risk of developing specific types of cancer and explore their sociodemographic characteristics. Another important dimension related to cancer patients is to explore the characteristics of their treatment therapy and to assess which treatment option is associated with better outcomes. This enhances the ability to optimize the therapeutic options offered to patients and improve their health outcomes.

Local detailed epidemiological studies enhance the ability to direct patient awareness programs for individuals who are at higher risk of developing cancer [2,3,4]. These directed programs educate individuals about the required lifestyle modifications to diminish their risk of developing cancer, such as smoking cessation, and improving dietary habits and lifestyle [5], which ultimately help in cancer prevention. Cancer registries enable the collection of data regarding the prevalence and incidence of different types of cancer and their associated death. Previous reports in Saudi Arabia have demonstrated an increase in the burden of cancer at the national level. Moreover, the relative incidence of different types of cancer has demonstrated a changing pattern, highlighting the need for up-to-date information to be readily accessible [1]. However, there are no studies that have explored the characteristics of cancer patients and the utilization of different cancer therapies in the southern region of Saudi Arabia. Knowing that the epidemiology of cancer differs significantly based on patients’ lifestyle, dietary habits, geographical location, and proximity to industrial zones and other variables that differ from one area to another in the same country [6,7], in addition to the large surface area of the Kingdom of Saudi Arabia (more than 2 million km^2^) and its geographical diversity [8], it becomes mandatory to conduct such types of studies in the southern region of Saudi Arabia to strengthen the ability to explore the epidemiology of the different types of cancer, identify patients at higher risk, and the characteristics of their treatment and act based on these data. The primary aim of this study is to explore the epidemiology of cancer, its characteristics, treatment patterns, and risk factors in the southern region of Saudi Arabia.

## 2. Methods

### 2.1. Study Design and Study Population

This was a retrospective cross-sectional observational study conducted using cancer patients’ medical records at Asir Central Hospital in the southern region of Saudi Arabia. Active patients’ records were extracted between January 2013 and December 2019. Active patients were defined as patients who were referred to the hospital regularly for follow-up, consultation, and receiving therapy (pharmacological and/or interventional therapy). Patients records were identified using the 10th version of the International Statistical Classification of Diseases (ICD_10_) system. Cancer cases were identified using neoplasm codes (C00–C96). Demographics data (age, gender, employment status, marital status, and income category) were extracted from patients’ medical records (paper-based). All data related to cancer diagnosis, type of treatment, and hospitalization (including the need for incentive care unit (ICU) admission, the duration of ICU admission, status upon discharge, stage of metastasis, drug use history, previous surgery, and having first degree relatives with cancer) were extracted from the computerized system of the cancer center. Data were extracted by registered pharmacy doctors and pharmacists.

### 2.2. Statistical Analysis

The descriptive analysis was reported as mean (+SD) for normally distributed quantitative and as median + IQR for non-normally distributed quantitative variables. Descriptive statistics were used to describe the participants’ demographic information. Demographic information was stratified further by gender and *p*-values are provided to indicate differences in variable distributions between men and women. Categorical data were reported as percentages and frequencies. Logistic regression analysis was used to identify predictors of ICU admission and death related to cancer. A confidence interval of 95% (*p* < 0.05) was applied to represent the statistical significance of the results, and the level of significance was assigned as 5%. SPSS (Statistical Package for the Social Sciences) Version 25.0 software (SPSS Inc., Chicago, IL, USA) was used to perform the statistical analysis.

### 2.3. Ethical Considerations

The study protocol was reviewed, and ethical approval was granted by the Research Ethics Committee of the Directorate of Health Affairs—Asir Region in Saudi Arabia (REC—1 September 2020).

## 3. Results

### 3.1. Patients’ Baseline Characteristics

A total of 2038 patients were identified during the study period with a mean age of 60.9 (SD: 19.0) years. More than half of them (50.9%) were men. The majority were single (67%) and with high income (69.9%). The majority (78.2%) were employed. More than half of them (57.1%) were non-Saudi. Around 69.9% had previous surgery, and 64% of the patients were smokers. The most common three diseases across the study sample were dyslipidemia, diabetes mellitus, and hypertension. Statin, inhaled corticosteroids, and heparin were the most commonly used chronic medications. For further details about the baseline characteristics of the study sample, refer to Table 1.

### 3.2. Epidemiology of Cancer

The patients had survived with their cancer for a median duration of 4 years (IQR: 2–6). Around 4.6% of the patients required ICU admission with a median period of 9 days (IQR: 5–14.75). Upon discharge, 61.6% of the patients’ cases were improved. The death rate during the study period was 10.9%. Around 20.8% of the cases were metastatic, of which 77.8% were at stage four of metastasis, and 19.7% of the patients were receiving chemotherapy for their disease (Table 2).

**Figure 1:**
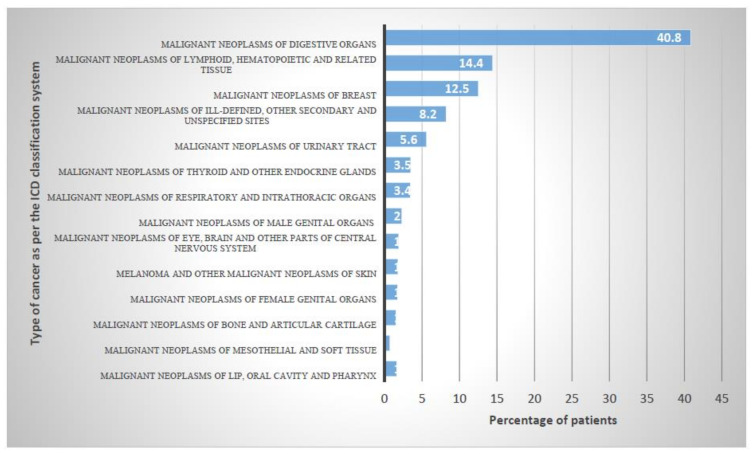
Epidemiology of cancer classified according to the ICD-10 code.

The most common types of cancers as per the ICD 10 system were “Malignant neoplasms of digestive organs” with 40.8%, followed by “Malignant neoplasms of lymphoid, hematopoietic and related tissue” and “Malignant neoplasms of breast” with 14.4% and 12.5%, respectively (Figure 1).

**Figure 2:**
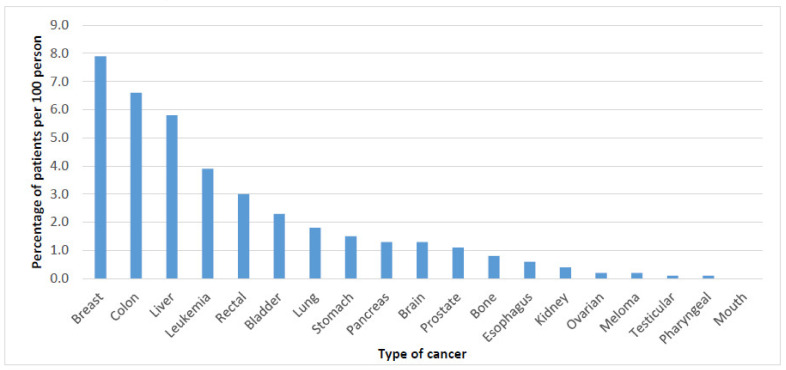
Type of cancer stratified by location.

**Figure 3:**
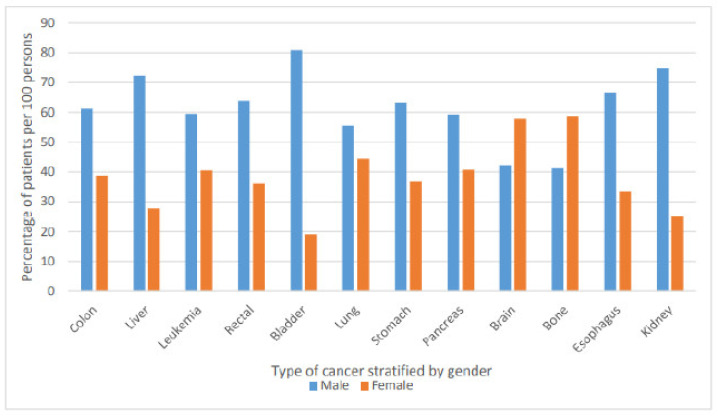
Type of cancer stratified by gender.

The most common three types of cancer were breast cancer, colon cancer, and liver cancer (Figure 2). Figure 3 below present the most common type of cancer stratified by gender.

### 3.3. Cancer-Related Death and ICU Admission Risk Factors

Older age (60 years and above) and using specific chronic disease medications (such as statins, inhaled corticosteroids, heparin, neuropathy treatment, angiotensin receptor blockers, diuretics, corticosteroids, and warfarin) were predictors associated with a higher risk of death due to cancer (*p* < 0.05). Smoking history, using specific chronic disease medications (such as inhaled corticosteroids, heparin, neuropathy treatment, angiotensin receptor blockers, diuretics, antiplatelets, corticosteroids, warfarin, and oral antidiabetic medications) and having previous surgery were predictors associated with higher risk of ICU admission (*p* < 0.05): refer to Table 3.

## 4. Discussion

This retrospective cross-sectional study assessed the epidemiology of cancer, its characteristics, treatment patterns, and the risk factors of cancer patients who attended Asir Central Hospital in the southern region of Saudi Arabia. It is the central hospital of the southwestern region and contributes to the treatment and management of most cancer cases in that region. This is the first study to investigate cancer epidemiology in the southern region of Saudi Arabia.

In our study, the most common three types of cancer were breast cancer, colon cancer, and liver cancer. All types of cancer were more common among men compared to women except for bone and brain cancer, which were more common among women. This was confirming in the findings of previous study that was conducted in Iraq, which concluded that most cancers were predominant in males [9]. Previous studies that examined the epidemiology of cancer and clinical characteristics of cancer patients were restricted to specific types of cancer or to ICU settings. A previous study that was conducted on critically ill cancer patients from 24 European countries has reported that 15% of the patients had a malignancy, 85% had solid tumors, and 15% had hematological cancer. Patients with solid cancers had the same severity of illness as the non-cancer population, but they were older, more likely to be a surgical admission, and had a higher frequency of sepsis. Patients with hematological cancer were more severely ill and more commonly had sepsis, acute lung injury/acute respiratory distress syndrome, and renal failure than patients with other malignancies; these patients also had the highest hospital mortality rate 58% [10].

Our results revealed a higher cancer rate among men (50.9%) versus women (49.1%). Such findings are in agreement with the numbers of the WHO report in 2018, which counted 12,263 new cases in men versus 12,222 new cases in women in Saudi Arabia. As the number of men in Saudi Arabia (19,212,443) is greater than that of the women (14,341,890), this suggests that the prevalence rate among women (0.085 per 100 persons) is higher than that for men (0.064 per 100 persons) [11]. Another Saudi Arabian study also reported that the total incidence rate of cancer in Asir was 58.6 versus 63.2 among men and women, respectively [1]. Furthermore, this study reported that individuals with a higher income demonstrated a higher rate of cancer (69.9%). Such results could be attributed to the changes that have happened over the past 30 years, where the lifestyle for higher-income individuals has become more sedentary, one which consumes higher levels of processed food and has become less active. All of these are contributing risk factors toward cancer [12]. Smoking is another risk factor that has been reported in the literature. Worldwide, smoking contributes to nearly 20% of cancer deaths [13]. Saudi Arabia has the fourth-highest consumption of tobacco worldwide; the prevalence of smoking among men is estimated at around 26.5%, whereas for women, it is 9% [14,15]; therefore, the association is justified.

In this study, we examined the epidemiological characteristics of cancer among individuals in the southern region of Saudi Arabia. Our findings showed a median duration of the disease of 4 years (IQR: 2–6) with 4.6% requiring ICU admission for a median period of 9 days (IQR: 5–14.75). Such findings implied an increased financial burden on the healthcare system. Cancer is increasingly evolving as a main public health concern and economic burden in Saudi Arabia [16,17]. However, cancer care in Saudi Arabia is based on the model of “find it and fix it”. Such a model lacks aspects of preventive measures that entail risk factor assessment and proactive intervention [18]. The findings of this study further explored that almost 20.8% of the patients reported metastasis, indicating later stages with more than 77.8% being in stage 4. Such results are alarming, as this will not only affect the economic burden on healthcare, including the direct cost of initial diagnostic and treatment cost, but also the survival rate and life expectancy of the individuals, as well as the long-term costs throughout survivorship. Although it varies according to cancer type and location, the 5-year survival rate is lower for patients with stage 4 cancer [19].

Interestingly, the findings showed that breast, colon, liver, leukemia, and rectal cancers are the top five cancers in the southern region of Saudi Arabia, respectively. The WHO report in 2018 highlighted how breast, colorectal, thyroid, leukemia, and non-Hodgkin lymphoma were the top five cancers in Saudi Arabia [11]. There is agreement in terms of some of them, but liver cancer was ranked the ninth overall in Saudi Arabia’s statistics. Hence, these results reveal that there is a variation among regions, particularly in Saudi Arabia, which is a large country. Asir is part of the southwest region; previous reports indicated that there, the prevalence rate of liver cancer was within the national average [20]. Two key risk factors contribute to the development of liver cancer. The first is chronic infection with hepatitis types B and C [21]. A study by Ayoola et al. reported a higher prevalence of hepatitis B in the southwest (5.4%) region [22], which includes Asir. The second risk factor is obesity. Several studies have reported the detrimental effect of obesity on liver cancer [12,23]. A study in 2007 reported that 33% of individuals living in Saudi Arabia were obese [23] and that the number had increased, which could explain the increase in liver cancer cases in Asir [21].

Risk factors associated with cancer-related death and ICU admission showed a positive association of death due to cancer with age. A recent study in Saudi Arabia indicated a steady increase in death due to cancer with age for individuals aged 69 years and below. However, a reduction in the death rate was observed among patients above the age of 70 years [12].

The presence of comorbidities affects the prognosis and survival of patients with cancer. Several research findings reported the effect of comorbidities on clinical management, the health outcome of cancer and cost, particularly in metastatic cancer patients [24,25]. Other studies have reported the association of comorbidities and delay of cancer detection and have even elicited a higher risk of complications [26,27]. Our results point to the direction of a statistically positive association between the use of cardiovascular medication (diuretics, Angiotensin receptor blockers, and statins), corticosteroids (oral and inhaled), and anticoagulants with death from cancer. Several studies have reported that patients with comorbidity have up to a 4 -fold increase in post-operative mortality rates in colon cancer [28,29,30]. Other findings have reported that patients with comorbidities usually will not follow standard cancer management and treatments, including surgery, chemotherapy, and radiation therapy. Furthermore, cancer patients with comorbidity may not complete the course of their cancer treatment. Post-operative complications and mortality are higher in patients with comorbidity [31,32,33,34,35,36].

Our study simultaneously evaluated the admissions to the ICU as an indirect implication of complications during the course of the treatment. ICU admissions were positively associated with previous surgeries, smoking, and comorbidities, particularly those related to cardiovascular events, diabetes, and the use of antiplatelets and anticoagulants and corticosteroids, both inhaled and oral. These results appear to be consistent with the trends of other research findings that patients with comorbidities demonstrated an increased risk of complications after surgical procedures for colon cancer, breast cancer, and lung cancer [25].

Further research is required on the prevalence of cancer stratified by type, gender, and other demographic characteristics in other areas in Saudi Arabia. This will help in identifying whether there are geographical differences between them and identify their underlying causes and risk factors.

### Strengths and Limitations

To the best of our knowledge, this is the first and largest study (2038 cancer patients) that has addressed the epidemiology of cancer, its characteristics, treatment patterns, and the risk factors of cancer patients in the southern region of Saudi Arabia. The data were collected from the primary source (i.e., medical records) and, hence, all the cases were captured with the review window. This study will contribute to the understanding of the status of cancer in the southwest area of Saudi Arabia, its characteristics, and potential risk factors. Being retrospective, we were able to capture issues that are rarely studied but can guide the cancer management planning process. We used ICD-10 codes to identify the study population, which increase the robustness of our inclusion criteria and confirm patients’ diagnosis. That said, it should be noted that the study has limitations. The study was conducted at a single center in the southern region of Saudi Arabia. However, Asir Central Hospital is the main center in the southern region receiving cases from all cities in the southern region. Duration of follow-up for the patients is not the same; for example, patients who were diagnosed in 2019 do not have 4 years of follow-up. This will increase the bias in the findings related to death estimates and ICU admissions as the patients do not have a similar duration of follow-up. Therefore, our findings should be representative of the Saudi population living in the southern region.

## 5. Conclusions

Several cancer risk factors were identified, including age, smoking, previous surgeries, and comorbidities. Breast, colon, and liver cancers were the most prevalent in the region. However, as with many other countries worldwide, Saudi Arabia faces the challenges of an increasing number of cancer cases and insufficient preventive measures, particularly for preventable cancers such as breast cancer. The results of this study should support decision-makers in the initiation of programs for key modifiable risk factors that enhance lifestyle changes and reduce cancer risks.

## Figures and Tables

**Table 1:** Patients’ baseline characteristics.

	Overall (*n* = 2038)		Men (*n* = 1038)		Women (*n* = 1000)		*p*-Value
Age at diagnosis (mean (SD)) (years)	60.9 (19.0)		62.0 (20.0)		59.7 (17.7)		0.007
Demographic variable	Frequency	Percentage	Frequency	Percentage	Frequency	Percentage	
Gender							
Men	1038	50.9					
Marital status							
Single	1365	67.0	612	59.0	753	75.3	0.000
Married	605	29.7	376	36.2	229	22.9	
Divorced	1	0.0	1	0.1	0	0	
Widowed	67	3.3	49	4.7	18	1.8	
Income category							
Low	133	6.5	59	5.7	74	7.4	0.000
Middle	481	23.6	205	19.7	276	27.6	
High	1424	69.9	774	74.6	650	65.0	
Employment status							
Student	27	1.3	16	1.5	11	1.1	0.000
Unemployed	368	18.1	128	12.3	240	24.0	
Employed	1594	78.2	874	84.2	720	72.0	
Retired	49	2.4	20	1.9	29	2.9	
Nationality							
Saudi	874	42.9	432	41.6	442	44.2	0.129
Non-Saudi	1164	57.1	606	58.4	558	55.8	
Previous surgery							
Yes	1425	69.9	770	74.2	655	65.5	0.000
Smoking history							
Yes	1304	64.0	693	66.8	611	61.1	0.004
Comorbidities							
Dyslipidemia	333	16.3	177	17.1	156	15.6	0.204
Diabetes mellitus	299	14.7	163	15.7	136	13.6	0.100
Hypertension	280	13.7	145	14.0	135	13.5	0.741
Neuropathy	180	8.8	93	9.0	87	8.7	0.079
Heart diseases	85	4.2	45	4.3	40	4.0	0.235
Liver diseases	59	2.9	27	2.6	32	3.2	0.121
Thyroid disorders	34	1.7	24	23.1	10	1.0	0.007
Asthma	26	1.3	15	1.4	11	1.1	0.275
Epilepsy	17	0.8	7	0.7	10	1.0	0.024
Arthritis	15	0.7	8	0.8	7	0.7	0.830
Tuberculosis	7	0.3	3	0.3	4	0.4	0.210
Depression/Anxiety	5	0.2	1	0.0	4	0.4	0.030
Nephropathy	3	0.1	2	0.2	1	0.1	0.176
GERD/Acid reflux diseases	3	0.1	1	0.1	2	0.2	0.543
Eye disorders	2	0.1	2	0.2	0	0	0.317
Drug use history							
Statin	613	30.1	296	28.5	317	31.7	0.064
Inhaled corticosteroid	258	12.7	99	9.5	159	15.9	0.000
Heparin	210	10.3	111	10.7	99	9.9	0.303
Neuropathy treatment	180	8.8	106	10.2	74	7.4	0.015
B-Blocker	179	8.8	87	8.4	92	9.2	0.283
Antipsychotic	163	8.0	92	8.9	71	7.1	0.083
Angiotensin receptor blocker	154	7.6	86	8.3	68	6.8	0.118
Diuretic	141	6.9	59	5.7	82	8.2	0.016
Antidepressant	139	6.8	67	6.5	72	7.2	0.281
Insulin	132	6.5	55	5.3	77	7.7	0.017
Nitrate	128	6.3	70	6.7	58	5.8	0.216
Angiotensin-converting enzyme	95	4.7	47	4.5	48	4.8	0.426
Antiplatelet	87	4.3	33	3.2	54	5.4	0.009
Corticosteroid	78	3.8	46	4.4	32	3.2	0.091
Ulcer treatment	58	2.8	23	2.2	35	3.5	0.053
Anticoagulant (warfarin)	50	2.5	29	2.8	21	2.1	0.193
Aspirin	46	2.3	30	2.9	16	1.6	0.034
Oral antidiabetic	43	2.1	24	2.3	19	1.9	0.311
Anxiolytic	42	2.1	11	1.1	31	3.1	0.001
Anticonvulsant	16	0.8	5	0.5	11	1.1	0.091
Calcium channel blockers	3	0.1	2	0.2	1	0.1	0.514

SD: standard deviation, GERD: gastroesophageal reflux disease.

**Table 2:** Characteristics of disease among the study sample.

Variable	Frequency	Percentage
Duration of disease (median (IQR) (years)	4.00 (2.00–6.00)	
Required ICU admission		
Yes	94	4.6
Duration of stay at the ICU (median (IQR) (days) (*n* = 94)	9.00 (5.00–14.75)	
Status on discharge from the inpatient department (*n* = 828)		
Improved	510	61.6
Transferred	135	16.3
Died	90	10.9
Stable	81	9.8
Unstable	12	1.4
Metastasis		
Yes	424	20.8
Metastasis stage (*n* = 424)		
Stage 1	2	0.5
Stage 2	33	7.8
Stage 3	59	13.9
Stage 4	330	77.8
Type of treatment		
Chemotherapy	402	19.7
Surgery	359	17.6
Radiotherapy	29	1.4
Combination therapy	1248	61.2
First degree relative with cancer history		
Yes	7	0.3
If yes, was it the same type of cancer that the patient suffers from? (*n* = 7)		
Yes	6	85.7

IQR: interquartile range, ICU: intensive care unit.

**Table 3:** Risk factors associated with cancer death and ICU admission.

Variable	Odds Ratio (95% CI) for Death	Odds Ratio (95% CI) for ICU Admission
Age		
(below 60 years) (Reference)	1.00	1.00
(60 years and above)	1.65 (1.05–2.59) *	1.01 (0.65–1.58)
Gender		
Women (Reference)	1.00	1.00
Men	1.05 (0.68–1.61)	0.97 (0.62–1.51)
Marital status		
Single (Reference)	1.00	1.00
Married	1.49 (0.76–2.90)	0.63 (0.36–1.09)
Divorced	-	-
Widowed	0.72 (0.28–1.86)	1.63 (0.78–3.38)
Income category		
Low (Reference)	1.00	1.00
Middle	0.41 (0.11–1.52)	0.56 (0.12–2.65)
High	2.43 (0.66–9.05)	1.78 (0.38–8.41)
Employment status		
Student (Reference)	1.00	1.00
Unemployed	1.94 (0.98–3.87)	1.11 (0.60–2.08)
Employed	0.32 (0.12–0.82) *	0.87 (0.43–1.75)
Retired	1.28 (0.52–3.16)	1.02 (0.35–3.02)
Previous surgery		
No (Reference)	1.00	1.00
Yes	1.04 (0.62–1.72)	2.91 (1.77–4.78) ***
Smoking history		
No (Reference)	1.00	1.00
Yes	0.69 (0.40–1.22)	2.05 (1.19–3.51) **
Drug use history		
Not using the medication (Reference)	1.00	1.00
Statin	2.01 (1.22–3.31) **	1.27 (0.79–2.06)
Inhaled corticosteroid	2.15 (1.39–3.35) **	3.31 (2.10–5.21) ***
Heparin	2.04 (1.28–3.24) **	2.03 (1.25–3.27) **
Neuropathy treatment	1.81 (1.10–2.96) *	1.93 (1.16–3.23) *
B-Blocker	1.35 (0.81–2.27)	1.49 (0.89–2.51)
Antipsychotic	1.45 (0.85–2.45)	1.58 (0.91–2.74)
Angiotensin receptor blocker	1.85 (1.10–3.09) *	2.25 (1.33–3.80) **
Diuretics	3.21 (1.99–5.19) ***	2.05 (1.19–3.51) **
Antidepressant	1.20 (0.67–2.17)	1.19 (0.64–2.19)
Insulin	0.96 (0.51–1.81)	1.56 (0.86–2.81)
Nitrate	1.28 (0.71–2.31)	1.68 (0.94–2.99)
Angiotensin-converting enzyme	1.21 (0.60–2.43)	1.33 (0.67 –2.64)
Antiplatelet	1.73 (0.92–3.27)	2.01 (1.07–3.79) *
Corticosteroid	1.97 (1.01–3.81) *	2.43 (1.22–4.85) *
Ulcer treatment	1.07 (0.45–2.58)	0.41 (0.13–1.34)
Anticoagulant (warfarin)	2.23 (1.04–4.77) *	2.40 (1.10–5.26) *
Aspirin	1.40 (0.57–3.39)	1.73 (0.69–4.33)
Oral antidiabetic	1.56 (0.64–3.82)	2.46 (1.07–5.64) *
Anxiolytic	0.96 (0.34–2.76)	1.37 (0.51–3.65)
Anticonvulsant	0.78 (0.18–3.50)	0.96 (0.22–4.30)
Calcium channel blockers	-	-

CI: confidence interval. * *p* < 0.05, ** *p* < 0.01, *** *p* < 0.001.

## Data Availability

Not applicable.
